# Mapping Effectiveness Studies of Occupational Therapy in Africa: A Scoping Review

**DOI:** 10.1155/2023/6688222

**Published:** 2023-11-22

**Authors:** Nicola Ann Plastow, Monique de Wit, Megan Brown, Monica de Kock, Patricia Pretorius, Saskia Pienaar, Wernice Venter

**Affiliations:** Division of Occupational Therapy, Faculty of Medicine and Health Sciences, Stellenbosch University, Cape Town, South Africa

## Abstract

**Background:**

Contextual relevance is an important consideration for evidence-based practice, especially in low- and middle-income countries where the nature of practice may differ from high-income countries. Resources and access to rehabilitation are constrained, and service-users face a range of intersecting challenges to activity and participation.

**Aim:**

To evaluate the body of evidence for the effectiveness of occupational therapy in Africa, and to determine if systematic reviews with meta-analysis and/or meta-synthesis are feasible. *Methods and Analysis*. We conducted a systematic scoping review of published and grey literature by following PRISMA-ScR guidelines across 13 databases and through personal contact with occupational therapists across Africa. Covidence software was used to manage a blind review process by at least three reviewers per included article. The McMaster Quantitative Review Form, NHMRC levels of evidence, the Cochrane PROGRESS-Plus health equity criteria, and the TIDieR checklist informed data extraction using Microsoft Forms.

**Results:**

The search yielded 4199 articles, of which 45 were included. Evidence in six fields of practice included paediatrics, mental health, physical rehabilitation, hand therapy, work practice, and community development, although the evidence was largely limited to South Africa (93% studies). Levels of evidence varied but included 13 RCTs. In all, 1957 participants were included, ranging in age from 25 days to 99 years, with a wide range of health conditions. Most studies reported a positive outcome for occupational therapy.

**Conclusion:**

Findings suggest a moderate body of evidence to support occupational therapy in Africa. Systematic review with meta-analysis, assessment of risk of bias, and in-depth analysis of specific areas of practice are now required. All effectiveness studies in occupational therapy should include measures of occupational performance or participation, minimum reporting standard checklists should be used more consistently, and effect sizes should be consistently calculated and reported in effectiveness research.

## 1. Introduction

The overarching role of occupational therapy in Africa is to promote participation [1]. Evidence-based practice (EBP) should be a foundation for enacting this role, since EBP is a key attribute of occupational therapy graduates [2]. Working in an evidence-based way means making rehabilitation decisions based on the experience of the service user (what works well for me), the experience of the occupational therapist (what works well for my service users), and the research evidence (what has worked well for other people). The nature of occupational therapy is also shaped by the context in which evidence-based services are provided. Nevertheless, systematic reviews are widely considered the gold standard for evidence-based practice and are the highest in the hierarchy of evidence.

Occupational therapy service delivery is already informed by a number of systematic reviews internationally, which have provided evidence of the effectiveness of occupational therapy across the lifespan. For example, Novak and Honan's [3] systematic review of paediatric occupational therapy for children with disabilities identified 75 systematic reviews and 54 randomised controlled trials that met their inclusion criteria. From these studies, 40 interventions were graded Green/Go, indicating sufficient evidence for implementation. Bennett et al.'s [4] systematic review and meta-analysis demonstrated moderate effectiveness of occupational therapy at home for people with dementia and their carers, across a range of outcomes. The economic value of occupational therapy in acute and subacute hospital settings was also demonstrated by Wales et al. [5], with five studies showing value for money. Meta-analysis of 16,718 patients in seven studies also showed that occupational therapy reduces readmission rates for adults hospitalised for surgical and general medical care, particularly if the intervention focused on the transition from the hospital to the home [6]. Finally, for adults with depression, Christie et al. [7] found strong evidence for the effectiveness of return-to-work interventions on depressive symptoms. These selected systematic reviews demonstrate the breadth of occupational therapy and suggest its effectiveness throughout life for people with a range of health conditions. However, none of these reviews specifically considered how the evidence was shaped by the context of the research.

Although van Vuuren et al. [1] explored the role and scope of occupational therapy in Africa, we were unable to find any previous systematic or scoping reviews of the effectiveness of occupational therapy in Africa or other low- and middle-income country (LMIC) regions. This is problematic because effectiveness studies conducted in high-income countries (HICs) are not always applicable to evidence-based practice in LMICs, for a number of reasons. First, the nature of occupational therapy in LMICs may be different. For example, in South America, social occupational therapy has emerged as an area of practice that focuses on the needs of people who experience structural barriers to participation. Social occupational therapists work to address these barriers and their consequences [8]. Occupational therapists from 11 African countries highlighted the challenges of working with very limited resources and the need for culturally appropriate occupational therapy and emphasized a focus on spirituality as a core component of service delivery [1]. Occupational therapy also often needs to be population-based or include groups in contrast to working individuals [1]. Second, occupational therapists in Africa face unique intersecting challenges. These include poverty and malnutrition, violence and political instability, stigma and discrimination against people with disabilities, and the need to understand cultural gender norms and their impact on participation [1]. Third is access to rehabilitation services. In Africa, the number of occupational therapists per population is eight times lower than the level recommended by the World Federation of Occupational Therapists [9]. This means that therapists have less time available per person than would typically be available in a HIC. Structural inequities in occupational therapy service provision in Africa are another potential source of difference between LMICs and HICs. Ned and colleagues found the occupational therapy workforce of 5180 registered with the Health Professions Council of South Africa in 2018 to be predominantly white (66%), under age 40 (67.7%), working in urban areas, and employed in the private sector (74.8%) [9]. This means that people in rural areas, those living on low incomes, and those who speak a different language to their therapist may experience inequitable access to occupational therapy services.

These contextual challenges mean that it is important for occupational therapists in Africa and other LMICs to have access to research evidence that is relevant to their context. Using interventions that have been shown to be effective in similar contexts may enhance the impact of occupational therapy services. As an emerging profession in Africa, it is also important to raise the profile of the profession. Evidence-based practice increases the credibility of occupational therapy in Africa because therapists will be able to demonstrate their commitment to using scientifically valid and reliable evidence to inform their decision making. In a resource-constrained environment, evidence-based practice also helps to maximise the use of available resources. For example, research evidence may inform optimal treatment frequency and duration of treatment or the likelihood of further functional improvement after an injury. This helps to reduce waste or harm. A final benefit of having better access to research evidence from Africa is that therapists will be able to engage more easily in continuing professional development that is the most relevant to their practice.

If a systematic review has not been done, scoping reviews can assist researchers to map the available evidence and then identify and define specific research questions for subsequent systematic reviews [10]. Scoping reviews can also promote evidence-based decision-making, by evaluating the available body of evidence. This means determining the level of confidence we can place in research findings, based on the quantity and quality of the available evidence. This includes the size of the body of evidence, the consistency of findings, and the type of research conducted within a hierarchy of evidence.

The aim of this study was to evaluate the body of evidence for the effectiveness of occupational therapy in Africa, to determine if systematic reviews with meta-analysis and/or meta-synthesis are feasible. These further reviews would need to evaluate the strength of the evidence, to enable occupational therapists and policymakers in Africa to make informed decisions about service delivery. The research question that guided our review was as follows: “What is the size, scope, and characteristics of the available research investigating the effectiveness of occupational therapy interventions in Africa?”

Using the PICOS acronym, we included participants of any age or diagnosis, any intervention provided by an occupational therapist or under the direction of an occupational therapist, any comparison, any outcomes, and any study design included in the National Health and Medical Research Council's (NHMRC) Levels of Evidence [11].

## 2. Methods

We conducted a systematic scoping review following the procedures described in the JBI Manual for Evidence Synthesis [12]. For reporting, we used the PRISMA extension for Scoping Reviews (PRISMA-ScR): Checklist and Explanation guidelines [13]. A review protocol was completed as part of an undergraduate research module and is available from the authors.

### 2.1. Eligibility Criteria

We included primary research that was published at any time, in any language, and in any written format (journal articles, research reports, dissertation, and thesis). Any intervention provided by, or under the supervision of, an occupational therapist in any African country was included, to people of any age, either with or without a specified diagnosis, in any setting and with any measured outcomes. Details of the criteria are indicated in Table 1.

### 2.2. Information Sources and Search Strategy

The following databases were selected with the assistance of an expert librarian, to maximise the likelihood of finding research conducted in Africa: Cochrane Library, PubMed, SciELO, Web of Science, Sabinet Reference, OTseeker, Africa-Wide Information, CINAHL, Academic Search Premier, Health Source: Nursing/Academic Edition, MEDLINE, and ERIC. We emailed all the heads of occupational therapy at universities in the 13 countries in Africa where occupational therapy training is provided, using our personal contacts and email addresses available on the World Wide Web. We also contacted the Research Committee of the Occupational Therapy Association of South Africa (OTASA) and individual researchers in our personal networks. Additionally, a hand search of the South African Journal of Occupational Therapy was performed, together with a review of the reference lists of included articles, to identify additional studies missed in the database search. The final search date was 30 April 2022. The details of the search for each database are included in Table 2.

### 2.3. Study Selection

The study selection team included two researchers who were appointed as associate professor (NAP) and lecturer (MDW) in the division of occupational therapy at Stellenbosch University and five undergraduate students completing a research method module. The findings from each database search were loaded to Covidence software for data management [14]. Once duplicates were removed by the software, two members of the research team independently screened titles and abstracts, then full texts within Covidence. Inclusion and exclusion criteria were applied during screening. The reasons for excluding full text were recorded as wrong study design, not an occupational therapy intervention, not research, study not conducted in Africa, and not the primary research. The conflicts were resolved through consensus by at least two of the three authors. During data extraction, the inclusion and exclusion criteria were also applied, and additional studies that had been incorrectly included were then excluded.

### 2.4. Data Collection Items and Extraction

A list of items for data extraction was developed collaboratively and included items from a variety of checklists. Two checklists were used in our evaluation of study designs. The McMaster Quantitative Review Form [15] was used to identify important study characteristics including title, authors, year of publication, country where research was done, journal of publication, study design, research aim, sample size and type of sampling used, outcomes used and reporting of their psychometric properties, statistical tests used and identified level of significance, and whether effect size was reported or not. We categorised the field of occupational therapy inductively, according to our experience of the field.

The NHMRC's Levels of Evidence [11] were used to classify the hierarchy of evidence available. Level I included a systematic review of randomised controlled trials (RCTs). Level II included RCTs. Level III included comparative studies such as pseudo-RCTs (Level III-1); studies with a concurrent control group (not randomised), cohort studies, interrupted time series (ITS) with a control group, and case control studies (Level III-2); and studies with a historical control, more than two single arm studies, or ITS without control (Level III-3). Level IV included case series, posttest studies, or pretest/posttest studies.

The generalizability of results to other populations was assessed using the Cochrane PROGRESS-Plus health equity criteria [16, 17]. This included the following 10 items: place of residence, any indication of race/ethnicity/culture/home language/religion, occupation, gender or sex, level of education, socioeconomic status, diagnosis, level of disability, HIV status, and marital status or living arrangements. For studies including children, occupation and level of education were considered reported if the authors reported the children went to school and their grade anywhere in the article. Living arrangements were considered reported if the study reported with whom the child lived.

The TIDieR checklist (Template for Intervention Description and Replication) [18] was used to extract data on the nature of the intervention and evaluate the quality of reporting. High-quality reporting enables replicability of an intervention. The 12-item checklist includes the name of the intervention, rationale, what (materials and procedures), person delivering the intervention, how, when, where and how much, how the intervention is tailored to individual needs, changes made during the trial, quality of planning, and fidelity of implementation.

To evaluate the consistency of the body of evidence, we first identified each outcome measure used, the measurement intervals used, and then the mean and standard deviation for the intervention and control groups at pretest, posttest, and follow-up.

The data extraction was completed using Microsoft Forms. Two authors piloted the use of Microsoft Forms with five articles, capturing data in duplicate, and then evaluating whether data had been consistently extracted. The included articles were divided between three authors based on available capacity. Data extraction was completed independently on allocated articles by each researcher. Queries or concerns were discussed in regular meetings throughout the time of data extraction. The final data set was checked for missing data and consistency of reporting by the first author.

As this was a scoping review, we did not complete a risk of bias assessment of the included studies. We did, however, analyse for publication bias and selective reporting within the studies.

### 2.5. Summary Measures

The size of the body of evidence was determined by counting the number of unique interventions evaluated; i.e., if a study was available as both a thesis and publication, it was only counted once. The type and quality of study design was determined by the frequency and percentage of publications at each of the NHMRC's levels of evidence. The quality of intervention reporting was determined by a two-point scoring system based on Plastow et al. [19] to indicate if each item on the TIDieR checklist was reported or not reported. The consistency of the findings and the quantity of evidence was determined by counting the number of studies that reported a positive result, no change, or a negative result. For generalisability, data on the characteristics of participants were summarised using frequencies and percentages. For age and sample size, we calculated the range and mean across studies where data was available.

## 3. Results

### 3.1. Study Selection

**Figure 1:**
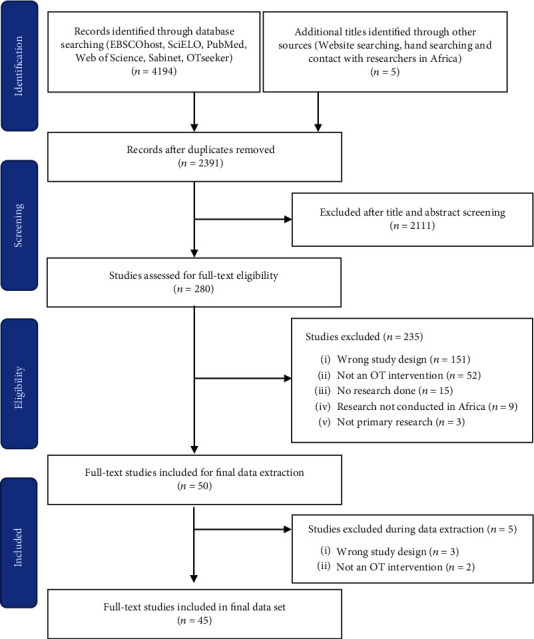
PRISMA diagram.

The study selection is presented in Figure 1. The initial database search yielded 4194 articles. An additional five articles were identified using other methods. When duplicates were removed, 2391 articles remained for screening. This suggests substantial overlap between the database findings and provides confidence that we identified most eligible studies. At title and abstract screening, we excluded 2111 studies. Thereafter, 280 studies were assessed for full-text eligibility. Of these, 230 were excluded, with reasons for exclusion indicated in Figure 1. When data extraction commenced, a further five studies were excluded.

### 3.2. Size and Scope of the Evidence

**Figure 2:**
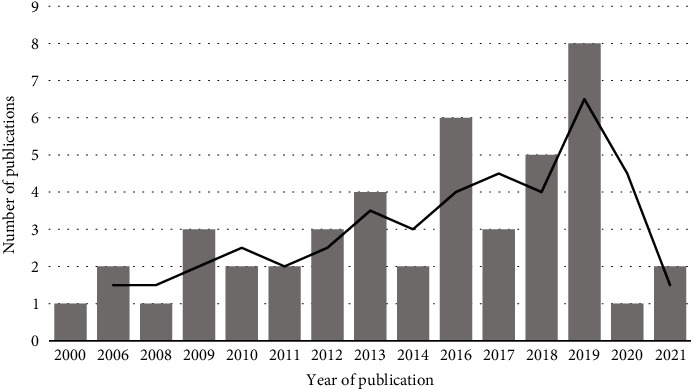
Number of publications by year (*n* = 45).

A total of 45 studies evaluating the effectiveness of occupational therapy were identified. The earliest publication was published in 2000. The overwhelming majority (80%, 36/45) were published in the past 10 years (see Figure 2). Published articles appeared predominantly in the South African Journal of Occupational Therapy (*n* = 19, 42%), with two articles published in the Journal of Hand Therapy and Occupational Therapy International. Eleven other articles (24%) were each published in different journals. Eleven studies were unpublished master's theses (24%).

Six fields of occupational therapy were identified among effectiveness studies: paediatrics (*n* = 19), mental health (*n* = 10), physical rehabilitation (*n* = 9), hand therapy (*n* = 4), work practice (*n* = 2), and community development (*n* = 1). One study was included in both the paediatrics and hand therapy categories as it included splinting for children with cerebral palsy (*n* = 1).

### 3.3. Levels of Evidence

A wide range of terminology was used to describe the study designs. We therefore classified each of the studies using terminology used in the NHMRC's levels of evidence [11] to enable comparison. Analysis using the NHMRC levels of evidence found no Level I study (systematic reviews of RCTs). At Level II, there were 13 randomised controlled trials, of which two were feasibility or pilot trials, and one used cluster randomisation. Level II evidence was only available in the fields of paediatrics (*n* = 7), mental health (*n* = 5), and hand therapy (*n* = 1). At Level III, there was 1 pseudorandomised controlled trial (Level III-1), 9 comparative studies with a concurrent control group (Level III-2), and 7 studies without concurrent controls (Level III-3). One-third of the studies (15/45) used a pretest, posttest, or case study design (Level IV). Most of these were physical rehabilitation (*n* = 5).  Table 3 illustrates the distribution of studies within the various fields of occupational therapy, as well as how they are represented under the levels of evidence.

### 3.4. Reporting of Participant Characteristics

A total of 1957 participants aged between 25 days and 99 years were included in the 45 studies. Using the PROGRESS-Plus criteria, participant characteristics were inconsistently reported, especially in journal articles. Of the 10 PROGRESS-Plus criteria, the authors reported a mean of 4.8 items (range 2-8, SD = 1.62).

Almost all of the research was conducted in South Africa (*n* = 42, 93%), with one study in Morocco, Uganda, and Namibia each. Most studies reported the location of the research within each country (40/45). Gender was similarly well-reported, with 40 of the 45 studies reporting the gender of participants. People receiving occupational therapy in Africa were almost evenly split between men (*n* = 722) and women (*n* = 771). There was, however, no report of any genders beyond the male/female dichotomy. Participants' health conditions were included in all 45 of the studies. However, the level of disability or functioning of participants was poorly reported, with only 12 studies reporting this equity characteristic.

People with a wide range of health conditions receive occupational therapy in Africa. The 20 studies within the field of paediatrics included children with cerebral palsy [20–24], visual perceptual difficulties [25], HIV [26], ADHD [27], learning disabilities [24, 28, 29], and very low birth weight [30]. Some studies were conducted with children without a diagnosis, but who were considered disadvantaged [31]; attended an ECD centre in rural Mahikeng, South Africa [32]; were aged 4 to 6 years [33] or in Grade R (the year before the start of formal schooling) ([34]); and had handwriting difficulties [35].

The ten studies in the field of mental health included people with mood disorders [36–41], psychotic disorders [36, 37, 40], conduct disorders [42], and substance dependence [43]. Two studies included participants without a mental health diagnosis. One addressed the needs of impoverished community-living people experiencing stress [44] and another teenagers at risk of alcohol use [45]. González-Bernal et al.'s [46] community intervention for people with disabilities included those with unspecified mental disabilities and emotional difficulties in Morocco.

People with physical disabilities were also included in the Moroccan study. The remaining participants in the nine studies using physical rehabilitation included people with stroke [47–51], diabetic foot ulcerations [52], traumatic brain injury [53], and upper and lower limb injuries [49]. In the field of work practice, one study included nursing auxiliaries working in a residential facility for people with intellectual disabilities [54] while another study included participants with traumatic brain injuries [55].

We identified five studies that evaluated the effectiveness of hand therapy, including children with cerebral palsy [56], people with mallet finger injury [57], flexor tendon injuries [58, 59], and crush injuries [60].

Other demographic characteristics were less frequently reported. Only 29% (*n* = 13) of the articles reported either race, ethnicity, culture, religion, or home language of the participants. Of these, three studies only included people who were white. Of the 23 studies that reported employment status, 12 included children in education. Of the remaining 11, only five studies reported the type of work by adult participants. These included being ECD practitioners; skilled, semiskilled, and unskilled occupations; nursing auxiliaries; unqualified/casual or professional; sedentary, light, medium, or heavy work; and maintenance worker. Similarly, levels of education and socioeconomic status of participants were poorly reported. Only nine studies (20%) reported the level of education of adult participants. Of these, only three studies included participants with any tertiary qualifications, and three studies included participants with only primary school or no formal education. Similarly, only 13 of the studies (29%) reported the socioeconomic status of the participants. Of these, 12 used a range of terminology to describe participants living on low incomes. One study was conducted in a private clinic, suggesting a higher socioeconomic status of participants. The only two studies that reported HIV status were Ramugondo's feasibility RCT to improve play of children who were HIV positive [26] and Msengana et al.'s intervention to improve upper limb mobility and personal management in people with stroke [51]. In addition, only four studies reported the marital status or living arrangements of participants.

### 3.5. Characteristics of Intervention Reporting

Studies reported between one and ten of the 12 TIDieR characteristics, with a mean and median of seven items reported per study. All of the studies provided a name for their intervention somewhere in the article. Interventions varied between multicomponent occupational therapy programs and specific treatment techniques. Most of the studies (*n* = 33) provided a rationale, goal, or theory informing the intervention. Less than half of the studies described how the interventions were tailored to the individual (*n* = 19). Only ten described any modifications made to the planned intervention, and only ten reported any plans to monitor intervention fidelity, although 14 studies provided at least some information on actual intervention fidelity.

### 3.6. Location of Intervention

Occupational therapy research was not limited to healthcare settings. Although 15 interventions were delivered in hospitals, five in specialist clinics (e.g., hand clinic and diabetic foot clinic), and three in rehabilitation centres, nine were delivered in the school setting, and one was delivered partly in participants' workplace. Two of the interventions were delivered in community venues such as the souk, Madrasa (Islamic school), and residential homes for older adults [46]. Only two interventions were delivered exclusively in participants' homes [21, 48], while another two included both healthcare facility and home-based components [50, 61].

### 3.7. Mode of Intervention

The mode of delivery, reported in 37 studies, also varied. An equal number of interventions were delivered in groups (*n* = 15) and one-to-one sessions with the occupational therapist (*n* = 16). Two studies included both individual and group intervention components [36, 49]. In a small number of studies, occupational therapists provided training and then supervised the intervention delivered by teachers and nurses [61–63]. One study used telehealth as the mode of delivery [48].

### 3.8. Dose

There was substantial variation in the duration and frequency of the interventions and the number of sessions offered by occupational therapists. Four studies evaluated the effectiveness of a single occupational therapy session [29, 38, 40, 41]. The remaining interventions took place over 1 week to 102 weeks (24 months). The mean intervention duration was 14 weeks, although the median was 8 weeks. Most interventions were delivered either daily (*n* = 19) or weekly (*n* = 18). Only 6 were provided monthly. However, there was little consistency when comparing duration and frequency. For example, daily interventions were provided over 1 to 52 weeks, while weekly interventions ranged between 2 and 8 weeks. The number of sessions varied between 1 and 150, with a mean of 22 sessions, and a median of 10 sessions.

### 3.9. Characteristics of Outcome Measures

Studies used a mean of 2.38 measures (range 1-9, SD = 1.64), although 18 studies only used one outcome measure. There was little consistency in outcomes used, even within the same field of practice. Less than half (21/45) of the studies included a measure of quality of life, participation, or occupational performance. The most commonly named outcomes at a participation level were the Barthel Index [47–50], level of creative ability using the Activity Participation Outcome Measure (APOM) or the creative participation assessment (CPA) [43, 52], Bayley Scales of Infant and Toddler Development [30, 64], and the Disability of the Arm, Shoulder, and Hand (DASH) [49, 60]. In two studies, researchers developed their own measures of participation [40, 62]. Few studies (12/45) reported translation of outcome measures or use of a version in an African language. Both the reliability and validity of the measures used were reported in a third of the studies (15/45).

### 3.10. Evidence of Effectiveness

Twelve of the 19 studies in paediatrics reported a positive effect on at least one outcome. Occupational therapy was reported to be effective in improving the functional performance of self-care; reducing caregiver support for social interactions; in-seat behaviour and attention to tasks; visual discrimination, spatial relations, and figure-ground perception; gross motor proficiency, running speed and agility, upper limb coordination, and strength; developmental progress; engagement; sensory processing; pencil grasp; physiological cost index and walking speed; and emotional intelligence.

Eight of ten studies in the field of mental health reported a positive effect on at least one outcome. These included the number of admissions, number of days in hospital, functional performance and participation (*n* = 5), and performance components (e.g., stress, mood, attention, concentration, and aggression). Function and participation were measured differently in all eight studies. One of the two studies in the field-of-work practice demonstrated an improvement in cognition [55]. In that study, seven of 10 participants returned to work.

All three studies investigating the effectiveness of hand therapy reported a positive outcome. Improvements were noted in the upper limb function in children with cerebral palsy [56], overall hand function and ability to work [60], and patient satisfaction [58]. Similarly, all eight of the physical rehabilitation studies demonstrated at least some level of effectiveness. Positive outcomes included improved functional independence [49, 50, 53], occupational performance [48], reintegration and creative participation [52], self-care [49, 51], mobility [49, 53], and knowledge of caregivers of people with disability [62].

## 4. Discussion

This study intended to map the evidence base for occupational therapy in Africa and to make recommendations for future systematic reviews. The 45 studies we identified suggest that future systematic reviews of the effectiveness of occupational therapy in Africa are feasible. This is particularly important to ensure that research findings are translated into health policy and practice [65]. The highest number of articles published was in the field of paediatrics, suggesting this is a good starting point. Six of these studies were RCTs, which suggests that a meta-analysis of outcomes may be feasible. The purpose of meta-analysis is to pool the results of a range of studies, to determine the overall effectiveness of an intervention. This requires a certain degree of similarity between the studies in their design, target population, outcome measures used, characteristics of the intervention, and methods of analysis [66]. Although the calculation of heterogeneity is beyond the remit of this scoping review, at face value, there are significant differences in the paediatric studies presented in Table 4. In addition, only high-quality studies with a low risk of bias should be included in a meta-analysis. This suggests that researchers would need to carefully consider their approach to meta-analysis, under the guidance of a biostatistician. For mental health, physical rehabilitation, and hand therapy, most international systematic reviews focus on specific diagnostic categories. The body of evidence is not yet large enough for meta-analysis in any of these three fields of practice. However, an in-depth narrative review of practice in mental health and physical rehabilitation services would provide useful information for clinicians establishing new services or enhancing existing ones.

After establishing the size of a body of evidence, it is important to consider the consistency of the findings. The most consistent findings of effectiveness were in the fields of physical rehabilitation and hand therapy. The effectiveness of occupational therapy for people with mental health problems was also demonstrated relatively consistently. In all three of these areas, there was a focus on measuring occupational performance or participation, which is where we would expect to see the greatest gains from occupational therapy. This contrasts with the effectiveness of occupational therapy for children and young people [1]. The evidence for this group of service users was much less consistent, with a third of studies demonstrating no effectiveness. However, this was also the group of studies that most frequently used only one outcome measure. In addition, many of the studies measured performance components, rather than occupational performance. Notably, none of the studies included a child-rated outcome measure like the Canadian Occupational Performance Measure [67] or the Child Occupational Self-Assessment [68]. Occupational therapy for older people is an important area of practice internationally that is supported by a strong body of evidence [69–71]. Our scoping review found that no studies focused exclusively on the needs of older people and no studies on the effectiveness of occupational therapy for people with dementia. Community development is a similarly important field of practice in Africa that is not being assessed for effectiveness.

The results of this scoping review also highlight a number of inequities in this body of evidence that limit the generalisability of the evidence base to healthcare systems across Africa. Studies to evaluate the effectiveness of occupational therapy were limited to four African countries. This is problematic since 15 African countries are now training occupational therapists for their local healthcare systems. These include Ghana, Kenya, Malawi, Mauritius, Nigeria, South Africa, Tanzania, Uganda, and Zimbabwe [72]. More recently, Cameroon, Ethiopia, Madagascar, Morocco, Namibia, and Rwanda have commenced programs. Race, ethnicity, and culture are contested social constructs that socially group people together according to how they look or the traditions they practice [73]. The failure to report race and ethnicity in occupational therapy research in Africa disregards the reality of inequity, injustice, and social stratification that is a consequence of the continent's colonial past and that has influenced population health. The low number of articles reporting race, ethnicity, culture, language, or religion also highlights the risk of inequity in occupational therapy service provision. Of the 13 articles that reported any of these constructs, almost a quarter (*n* = 3) only included white participants. Because these descriptors may hide health disparities, and limit the generalisability of findings, it is important to report these participant characteristics at this time [73].

The least reported health equity characteristic across the studies was HIV status. This is despite the fact that Africa is the continent with the largest number of HIV-positive people [74]. The importance of this equity characteristic was highlighted by Philip et al.'s qualitative study of the lived experience of people with disabilities and HIV in accessing HIV services [75]. That study found that HIV status, disability, stigma, poverty, and gender “collide in a hierarchy of identities to impede accessibility to HIV services” [75]. It is expected that access to occupational therapy services and the effectiveness of those services will similarly be impeded by the intersection of a range of different health equity characteristics. For example, De Klerk et al. [76] found that male gender, being unemployed, and receiving state-subsidised healthcare because of low income were some of the factors predicting nonattendance at a regional rehabilitation centre in Cape Town, South Africa [76]. This is why the limited reporting of socioeconomic status in the identified studies is also problematic. A number of the PROGRESS-Plus health equity characteristics point to the economic resources that may be available to a person that enable them to access healthcare. These include people's level of education, employment status, type of work, and level of income. None of these were adequately reported.

## 5. Conclusion

Evidence-based practice is a core attribute of occupational therapy graduates (Nicola Ann [2]). The findings from this review indicate emerging contextually relevant evidence to support the effectiveness of occupational therapy in Africa. A number of studies across the scope of occupational therapy practice have been conducted. More research is needed before making a judgement about the strength of this evidence. Future research should include a systematic review with meta-analysis of study findings, assessment of risk of bias, an in-depth analysis of assessments and interventions used in specific areas of practice, and an interrogation of the reasons why effectiveness is not always demonstrated. It is also recommended that all effectiveness studies in occupational therapy include a measure of occupational performance or participation, that minimum reporting standard checklists are consistently used to improve the quality of reporting, and that effect sizes are consistently calculated and reported in effectiveness research.

## Figures and Tables

**Table 1:** Inclusion and exclusion criteria.

Study characteristics	Included	Excluded
Participants	Any age or diagnosis Receiving treatment in an African country	No African country included
Intervention	Provided by an occupational therapist or under the supervision of an occupational therapist	No evidence of occupational therapy
Comparison	None or any	n/a
Outcome	Any form of outcome demonstrating the effect or effectiveness of occupational therapy	No measurement of outcomes
Study design	Any study that used statistical methods to evaluate the effectiveness of an intervention	Qualitative research
Publication date	Any	n/a
Language	Any	n/a
Publication status	Peer-reviewed articles, unpublished postgraduate research reports, dissertations, or theses	Expert opinion

**Table 2:** Database search information.

Database	Search string	Limits
Cochrane Library	(occupational therapy OR occupational therapist OR OT) AND (effectiveness OR effect OR success^∗^ OR efficacy OR efficiency OR benefit OR influence OR outcome OR result OR improve^∗^ OR evidence OR significance OR implication OR value OR consequence^∗^ OR impact OR usefulness OR qualit^∗^ OR importance OR insight^∗^ OR barrier^∗^ OR challenge^∗^ OR problem) AND (Africa)	None
PubMed		
SciELO		
Web of Science		
Sabinet Reference		
OTseeker		
EBSCOhost: Africa-Wide Information, CINAHL, Academic Search Premier, Health Source: Nursing/Academic Edition, MEDLINE, and ERIC		

**Table 3:** Levels of evidence for occupational therapy in Africa.

OT field of practice	Level of evidence					
	II	III-1	III-2	III-3	IV	Total
Paediatrics	7		6	2	4	19
Mental health	5		2	1	2	10
Physical rehabilitation		1	1	1	5	8
Hand therapy	1			1	2	4
Work practice					2	2
Community development				1		1
Paediatrics and hands				1		1
Grand total	13	1	9	7	15	45

**Table 4:** Effectiveness studies from Africa by field of practice and level of evidence.

Area of practice							
Authors	Study aim	Level of evidence	Sample size	Name of intervention	Outcome measures	Effective	Size of effect
Community development							
González-Bernal et al. [46]	The aim of this research was to verify the effectiveness of a community-based OT intervention in the volition, quality of life, and perceived self-stigma of people with disabilities in the Moroccan city of Azrou.	III-3	94	Community occupational therapy	Volitional questionnaire, WHOQOL-BREF, the stigma consciousness questionnaire, attribution questionnaire, ad hoc interview (Likert scale)	Yes	Not reported

Hands							
Naudé and de Klerk [76]	To explore aspects of feasibility related to the recruitment, consent, and retention rates in consideration of a future definitive randomised control trial. In addition, preliminary results of an early active therapy protocol compared to an early passive therapy protocol were reported on, together with patient satisfaction and demographics.	II	31	Early active mobilisation	Total active motion, Jamar hand dynamometer, Michigan Hand Questionnaire, Smith Hand Function Evaluation	Yes	Not reported
Spark, et al. [59]	To determine the range of movement (ROM), power, and pinch grip strength post-FTR and to establish factors that may affect these	III-3	126	Passive motion, controlled active motion, or early active motion protocols	Bilateral hand ROM, bilateral grip strength, and pinch strength	No	Not reported
Young et al. [60]	To report on the rehabilitation and functional outcomes of a patient who had his hand replanted at the distal forearm in a private practice setting in Umhlanga, South Africa	IV	1	Early active mobilisation	ROM—goniometer; sensation—monofilaments and disk criminator; muscle strength—hydraulic dynamometer, B&L pinch gauge, and manual muscle testing; hand function—nine hole peg test, Jebsen-Taylor Hand Function Test, and DASH	Yes	Not applicable
Devan [57]	To describes a technique that utilizes a combination of an orthosis and Kinesio tape, thereby creating a treatment protocol that shortens the immobilisation phase for these patients	IV	16	Early active mobilisation	Goniometer	Yes	Not reported

Mental health							
Gandawa [45]	To determine whether participation in an eight-week volunteer program for learners was more effective than an eight-session life skill training program in improving alcohol use behaviour and perceptions	II	34	Volunteer program vs. life skill group	Alcohol expectancy questionnaire—adolescents, alcohol timeline follow-back	No	None
Strauss et al. [40]	To determine the effect of the tempo of music on the activity participation of the inhibited and agitated mental healthcare users with acute psychosis	II	160	Music tempo	Researcher developed their own assessment of participation because “there are no assessments to measure participation”; included attention, following of instructions, directedness towards activity, and willingness to participate	Yes	Not reported
Van Rensburg et al. [42]	To determine the short-term effect of a group drumming intervention program on aggression among adolescent girls at a school for girls diagnosed with conduct disorder in Bloemfontein in the Free State	II	26	Drumming program	Demographic questionnaire, aggression scale	Yes	Not reported
De Villiers [41]	To determine the effect of a 90-minute online intervention, to improve knowledge of individualized sensory processing preferences and associated modulation strategies on the occupational performance of adults with major affective disorders	II	27	Education on individualized sensory processing styles and associated modulation strategies	Canadian Occupational Performance Measure, WHODAS 2.0, PHQ-9, and GAD-7	Yes	Not reported
Crouch [44]	To make an effective stress management program available to the impoverished rural community of the Acornhoek area of the Limpopo province	II	120	Stress management program	Questionnaire 1, questionnaire 2—both developed from literature and contextualized	Yes	Not reported
Engelbrecht et al. [36]	To determine whether attendance at an occupational therapy-led day treatment centre for mental healthcare users affects the use of inpatient services in South Africa	III-2	44	Occupational therapy-led day treatment centre	Number of admissions, days in hospital	Yes	Medium to large
Ramano and de Beer [39]	To compare the outcomes of the proposed new occupational therapy (SCO) group program, with the existing occupational therapy (SCN) group program	III-2	100	New occupational therapy (SCO) group program	Bay Area Functional Performance Evaluation-Revised (BaFPE-R)	Yes	Very small to medium
Grobler [37]	To compare an activity program with an outcome-based program for optimal functioning and reintegration of mental healthcare users into the community	III-3	102	Activity-based occupational therapy vs. outcome-based occupational therapy	International Classification of Functioning, Disability and Health	Yes	Not reported
Plastow et al. [38]	To examine the effects of an occupational therapist-led African drumming group on mental well-being among adult psychiatric inpatients with mood disorders	IV	13	Occupational therapy-led African drumming	Stellenbosch Mood Scale (STEMS), Enjoyment of Interaction Scale, Patient Health Questionnaire-9 (PHQ-9), Generalized Anxiety Disorder-7 (GAD-7)	Yes	Large
Silaule and Casteleijn [43]	To evaluate the change in activity participation of mental healthcare users attending an occupational therapy program	IV	64	Occupational therapy program	Activity Participation Outcome Measure (APOM)	Yes	Not reported

Paediatrics							
Vlok et al. [25]	To investigate the effect of ocular motor exercises, in combination with a visual perceptual program, on the visual perception of seven-year-old learners with visual perceptual problems	II	32	Visual perceptual intervention program	The Developmental Test of Visual Perception, 2nd edition, by Hammil, Pearson, and Voress 15 (DTVP-2) was used as the pre- and postprogram measurement	No	Small
Ramugondo et al. [26]	To describe the playfulness in children with HIV and PHE on HAART living in challenging socioeconomic areas in South Africa, and to evaluate the feasibility and preliminary effectiveness of a play-informed, caregiver-implemented, home-based intervention (PICIHBI) for improving play	II	24	Play-informed, caregiver-implemented, home-based intervention (PICIHBI)	Test of playfulness, background information questionnaire	No	Small
Hughes et al. [22]	To determine the effect of a soft neoprene thumb abductor splint on upper limb function in children with CP with a thumb-in-palm deformity	II	28	Neoprene thumb abduction splints and home program	Quality of Upper Extremity Skills Test	No	Very small to large
Buckle et al. [27]	To determine whether learners with attention deficit hyperactivity disorder (ADHD) and definite difference in sensory processing would be able to improve their in-seat behaviour, task completion speed, and attention-to-task as a result of a weighted vest	II	30	Weighted vests	Time seated per 20 minutes (in-seat behaviour), time in seconds (task completion), Conners' Continuous Performance Test II (CPT II) (attention to task)	Yes	Not reported
Hewson [61]	To design and evaluate a postural control program for infants, in which defined stimulatory activities are embedded into the everyday activities of the parents	II	50	Infant postural control program (IPCP)	Screening Questionnaire, Baby's Day Diary©, Peabody Developmental Motor Scales–2nd Edition (PDMS-2)	Yes	Not reported
Lecuona et al. [30]	To investigate the effect of Ayres Sensory Integration (ASI) on the development of premature infants in the first 12 months of life	II	24	Ayers Sensory Integration (ASI)	the Infant/Toddler Sensory Profile (ITSP), the Test of Sensory Function in Infants (TSFI), and Bayley III Scales of Infant and Toddler Development (BIII)	Yes	Not reported
Van Heerden et al. [34]	To establish whether a life skill program would have a short-term effect on the emotional intelligence of children in Grade R	II	88	Life skills	Cilliers Emotional Intelligence Test	Yes	Not reported
Russell et al. [64]	To assess the effectiveness of the Developmental Resource Stimulation Programme for children with DS younger than 42 months	III-2	30	Developmental Resource Stimulation Programme	The Bayley Scales of Infant and Toddler Development, 3rd edition (Bayley Scales III)	No	Not reported
Levin [29]	To identify whether Stetro pencil grips, inclined surfaces, and splints are effective in enhancing writing speed and legibility in grade two children with learning disabilities	III-2	22	Assistive devices for pencil grasp	Author-developed handwriting assessment including speed and legibility (letter formation, spacing between words, letter spacing between lines, accuracy, and general appearance)	No	Not reported
Chedzey [31]	To determine the level of efficacy of the CTP on the development of three-to-four-year-old children in the rural Mahikeng areas	III-2	49	The cross-trainer program (CTP)	ECDC (Early Childhood Development Criteria) consisting of Section A: Cognitive SRRA (School Readiness Risk Areas), Section B: Fine Motor Coordination, and Section C: Gross Motor Coordination	Yes	Not reported
Salzwedel [33]	To determine the effect of the SEMOSTI sensory-motor program, on the gross motor proficiency of four-to-six-year-old children	III-2	73	SEMOSTI (sensory motor stimulation program)	Bruininks-Oseretsky Test of Motor Proficiency, Second Edition (BOT-2), questionnaires: physical activity (frequency, duration, type in domains of vigorous, low to moderate, and sedentary)	Yes	Not reported
Smit [35]	To determine the effect of a classroom-based program on handwriting for children with pencil grasp problems	III-2	45	Pencil grip provided	Lyytinen-Lund (1998) observation schedule, pinch meter, Minnesota Handwriting Assessment	Yes	Large
Hamer-Rohrer et al. [21]	To establish whether a CIMT (constraint-induced movement therapy) course in the home environment will result in improved hand function and performance in functional skills (activities of daily living) in a young child with asymmetrical cerebral palsy	III-3	1	Constraint-induced movement therapy	Pediatric Evaluation of Disability Inventory	Yes	Not reported
Du Plessis et al. [23]	To determine the effect of 12 hippotherapy sessions on physiological effort and walking speed over 60 m	III-3	10	Hippotherapy	Physiological cost index (PCI) and walking speed	Yes	Not reported
Russell et al. [24]	To establish the effect of combined therapy approaches (CTA) on the intervention of four children aged 48.1 months to 60 months with cerebral palsy (CP)	IV	4	CTA (combined therapy approaches)	PEDI-CAT, gross motor function measure, and goal attainment scale	No	None
Demopoulos [28]	To investigate the relationship between a sensory diet and in-seat behaviour of a learner in the classroom	IV	12	Sensory diet	Daily behaviour assessment scale	No	None
Bastable et al. [20]	To determine the effect of a nonpowered, self-initiated mobility program on the engagement of young children with severe mobility limitations	IV	4	Self-initiated mobility program	The Individual Child Engagement Record—Revised (ICER–R)2, Gross Motor Functioning Classification Level (baseline only)	Yes	Medium to large (Per participant)
Jorge et al. [63]	To explore whether 7-to-24-month-old infants with fussy behaviour would respond to an intervention program consisting of a two-week sensory diet and parent education	IV	12	Parent education and sensory diet	Demographic questionnaire, survey questions about the parents' knowledge of RSPD, parent self-report Infant-Toddler Symptom Checklist	Yes	Large
Delgado [56]	To investigate the effect a supination splint on the upper limb function of children with cerebral palsy for six months after they were injected with botulinum toxin A	III-3	10	Supination splint	Modified Ashworth scores; goniometry measurements of the elbow, forearm, wrist, and thumb; the Quality of Upper Extremity Skills Test (QUEST) and a subjective hand assessment	Yes	Not reported

Physical rehabilitation							
Jansen and Casteleijn [52]	To investigate whether occupational therapy, tailored to the level of motivation for patients with diabetic foot complications, has more positive treatment outcomes than occupational therapy that is not	III-1	10	Occupational therapy program using Vona du Toit Model of Creative Ability	Reintegration to normal living index, wound tracing, creative participation assessment	Yes	Not reported
Kamwesiga et al. [48]	To evaluate the feasibility of (i) a mobile phone supported family-centred ADL intervention F@ce™ and (ii) the study design for evaluating the effects of the intervention on the perceived impact of stroke, perceived participation in everyday life, and self-efficacy in everyday activities among persons with stroke and their families in Uganda	III-2	30	F@ce	COPM, Self-Efficacy, Stroke Impact Scale 3.0 Uganda version, Barthel Index, Occupational Gaps Questionnaire Ugandan version	Yes	Varies, but favours F@ce
Msengana et al. [51]	To establish the profile of the dysfunction with which stroke survivors present as well as the recovery their upper limb movement and independence in personal management on referral, at discharge and for two months postdischarge	III-3	59	Occupational therapy	The Fugl-Meyer Assessment Upper Extremity (FMA UE) and the South African Data Functional Medicine (SADFM) Beta Scale®	Yes	Not reported
Mamabolo et al. [50]	The aim of this study was to establish the degree of functional independence of patients with stroke at discharge and more than six weeks postdischarge	IV	68	Stroke group and home-based intervention	Barthel Index	Yes	Not reported
Haffejee et al. [53]	To determine the functional mobility outcome and the factors that influence it post-TBI at discharge from hospital	IV	60	Occupational therapy	Functional Mobility Outcomes from the Rivermead Mobility Index Score	Yes	Not reported
Cawood and Visagie [47]	To describe the functional outcomes achieved by stroke survivors in an urban Western Cape province	IV	53	Occupational therapy	Stroke Impact Scale (SIS) Version 3.0, Modified Barthel Index (MBI)	Yes	Not reported
Graham [62]	To determine the value of CBR training, undertaken by the Department of Occupational Therapy	IV	20	Child care course	Questionnaire on knowledge gained from each workshop, retention of knowledge over one month, and skills learned used in daily handling of children	Yes	Not reported
Kloppers [49]	To describe the outcomes achieved by clients after participating in rehabilitation at the Bishop Lavis Rehabilitation Centre (BLRC) over a three-month period	IV	78	Rehabilitation (occupational therapy, physiotherapy, and limited speech and language therapy)	Adapted Zambian Activity and Participation questionnaire, Barthel Index, Oswestry Back Pain questionnaire, AIMS2-SF Arthritis Impact Scale, DASH (Disability of the Arm, Shoulder, and Hand), clinical mobility scale, carer stress scale, and CRALM	Yes	Not reported

Work practice							
Van der Linde [54]	To determine the impact of a staff development program to increase knowledge, skills, and attitudes to work with PIMD on their perceived job satisfaction over time	IV	12	Staff training	Job satisfaction questionnaire	No	Not reported
Soeker [55]	To determine whether MOOSE is an effective model to enhance the cognitive skills of individuals with brain injury	IV	10	MOOSE (Model of Occupational Self Efficacy) program	Montreal Cognitive Assessment	Yes	Not reported

## Data Availability

A review protocol was completed as part of an undergraduate research module and is available from the authors. The original data from this review are available from the corresponding author on request.
